# Perceived indoor thermal environment and depressive symptoms among older adults in the Japan Gerontological Evaluation Study

**DOI:** 10.1038/s41598-025-15922-9

**Published:** 2025-08-22

**Authors:** Maho Iwata, Anna Kinugawa, Masamichi Hanazato, Katsunori Kondo, Ken Osaka, Kenji Takeuchi

**Affiliations:** 1 Tohoku University School of Medicine, Miyagi, Japan; 2 Department of International and Community Oral Health, Tohoku University Graduate School of Dentistry, Miyagi, Japan; 3 Department of Environmental Preventive Medical Sciences, Center for Preventive Medical Sciences, Chiba University, Chiba, Japan; 4 Department of Community Building for Well-Being, Center for Preventive Medical Sciences, Chiba University, Chiba, Japan; 5 Research Department, Institute for Health Economics and Policy, Tokyo, Japan; 6 Division of Statistics and Data Science, Liaison Center for Innovative Dentistry, Tohoku University Graduate School of Dentistry, Miyagi, Japan

**Keywords:** Housing, Indoor environment, Indoor cold, Indoor heat, Depression, Older adults, Epidemiology, Epidemiology, Risk factors

## Abstract

Depression is a major global health problem and presents a significant health burden. Although abnormal indoor temperatures are known to be associated with adverse health effects, their link to depression is unclear, especially regarding indoor heat. This study aimed to examine the association between perceived indoor cold or heat and depressive symptoms among Japanese older adults. We used cross-sectional data from the 2022 Japan Gerontological Evaluation Study (JAGES), targeting independent older adults aged ≥ 65 years. The prevalence of depressive symptoms was the dependent variable, while the participants’ self-reported ability of their housing to prevent indoor cold or heat was the independent variable. Prevalence ratios (PRs) and 95% confidence intervals (CIs) were estimated using Poisson regression models with potential confounders as covariates. Additionally, we conducted a stratified analysis by geographical regions to explore regional differences. Of a total of 17,491 participants (49.4% male), 22.8% reported depressive symptoms. After adjusting for confounders, participants living in houses that could not prevent cold or heat had a 1.57 (95% CI = 1.45–1.71) times higher prevalence of depressive symptoms than those living in houses that could prevent cold or heat. In the stratified analysis by geographical regions, a significant association was observed in all areas except for Hokkaido, the northernmost area with the coldest climate. In conclusion, perceived indoor cold or heat was associated with an increased prevalence of depressive symptoms among older adults. Further research is expected to investigate the impact of improving the indoor thermal environment, such as by installing insulation, on the prevention of depression.

## Introduction

Depressive disorder is a major global health problem^1^, ranked as the second leading cause of years lived with disability (YLDs) and the 12th leading cause of disability-adjusted life-years (DALYs) according to the 2021 Global Burden of Disease Study^2^. Depression in older adults is known to increase the risks of dementia, anxiety, suicide, frailty, and mortality, as well as cognitive and functional impairments^3–5^. Therefore, depressive disorder presents a significant health burden, and its prevention is crucial for addressing an aging society.

Recently, there has been growing evidence that indoor cold and heat exposures are associated with adverse health effects^6,7^. As awareness of the importance of the living environment is increasing, the World Health Organization (WHO) issued Housing and Health Guidelines in 2018, highlighting the health risks associated with abnormal indoor temperatures and recommending a minimum indoor temperature of 18 °C as well as protection from excessive indoor heat^8^. For example, low indoor temperatures are associated with increased risks of cardiovascular and respiratory diseases^9–12^, poorer sleep quality^13^, reduced physical performance^14^, and lower self-rated health^7,15^, whereas high indoor temperatures have been associated with sleep disturbance^16^, increased hospital admissions, and higher mortality rates^17^. Regarding mental and general health, perceived cold (which is a subjective measure) has been reported to increase risks of psychological distress^18^ and affect quality of life (QOL)^19^. Moreover, high indoor temperatures have been demonstrated to affect psychological distress and QOL^20^. Considering that climate change will continue to be one of the major global problems, and increased outdoor temperatures caused by global warming are known to increase mental health issues such as suicide, aggression, and psychological distress and exhaustion^21^, further studies on the health effects of indoor heat are needed. However, evidence of the effects of indoor cold and heat exposures on specific psychiatric diseases such as depression is still lacking, especially in terms of perceived heat.

The indoor thermal environment continues to be a serious issue in Japan. Despite many regions in Japan having four distinct seasons, an estimated 39% of existing houses are uninsulated^22^. In addition, heating in Japan is mostly intermittent and typically only used in certain rooms such as living rooms and bedrooms. This is evident from the fact that the amount of energy spent on heating is only one-quarter of that in European and American countries, where continuous whole-home heating is standard^23^. Perhaps for these reasons, more than 90% of Japanese households do not meet the WHO-recommended minimum indoor temperature of 18 °C in winter^24^, which could be inferred to lead to various health problems. Indoor heat in Japanese houses during summer is also becoming a serious issue due to rising outdoor temperatures caused by climate change and insufficient insulation^25^. In 2024, more than 90,000 people were transported by ambulance due to heat stroke, 38% of which occurred inside housings^26^, highlighting the need to address not only indoor cold in winter but also indoor heat in summer. Moreover, Japan has been experiencing a super-aged society since 2007. Considering that depression in later stage of life increases the risks of dementia, anxiety, frailty, and mortality as mentioned earlier^3–5^, and seniors aged over 60 years spend an average of 78% of their time at home^27^, it is necessary to explore the effects that this issue may have on depression among Japanese older adults. This issue is particularly relevant for functionally independent older adults, who have the ability to make decisions about their own living environment, unlike functionally dependent older adults who typically live in designated facilities.

This study focuses on community dwelling, functionally independent older adults in Japan and explores the hypothesis that exposure to perceived indoor cold or heat is associated with a higher prevalence of depressive symptoms.

## Methods

### Setting and participants

**Fig. 1:**
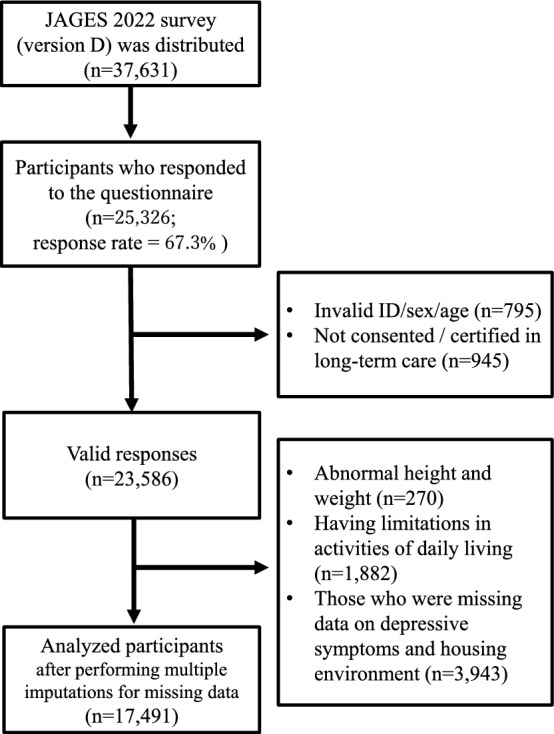
Flow diagram of the study participants’ selection.

This cross-sectional study was based on a self-reported questionnaire. We obtained data from the Japan Gerontological Evaluation Study (JAGES) conducted in 2022. The JAGES targeted community-dwelling older adults, aged ≥ 65 years, who were ineligible for long-term care insurance benefits, spanning 71 municipalities in Japan^28^. Eight questionnaire modules were randomly distributed to cover a wide range of topics, including community resources and support (module A), medical services and medication (module B), life after disaster and COVID-19 (module C), oral health and nutrition (module D), housing and daily life (module E), living environment and life space (module F), physical activity and optimism (module G), and activities of daily living and pain (module H). Among these eight modules, we used module E. The questionnaires were distributed and retrieved via mail. Exclusion criteria included participants who did not provide consent, those with invalid or missing data on sex, age, height, or weight, those certified for long-term care, those with dependent activities of daily living (ADL), and those with incomplete data on housing environment or depressive symptoms. After applying these exclusion criteria and performing multiple imputations for missing data, 17,491 participants were selected for analysis (Fig. 1).

### Dependent variable

We used the prevalence of depressive symptoms as the dependent variable. Depressive symptoms were measured using the Geriatric Depression Scale 15 (GDS-15), which is a validated tool for screening depression in older adults. The GDS-15 is assessed using 15 items, including questions such as “Are you basically satisfied with your life?”, “Have you dropped many of your activities and interests?”, “Do you feel that your life is empty?”, and “Do you often get bored?”^29^ Scores range from 0 to 15, with scores of 5 or higher indicating depressive symptoms and scores of 4 or lower indicating no depressive symptoms^30,31^. The GDS-15 is in the public domain, and permission for its use is not required. More information is available at the following link: https://web.stanford.edu/~yesavage/GDS.html

### Main exposure

Whether the participants’ housing could prevent heat or cold was used as the independent variable. From the multiple-selection question “Please choose all difficulties you are facing regarding your housing environment”, the option “Unable to prevent heat or cold” was used to assess whether the participants were living in cold or hot homes. This type of subjective indicator allows participants to respond based on their needs, and reflects the combined effects of indoor temperature, housing conditions, and personal preferences^18^.

### Covariates

Based on previous studies, demographic factors, health status, socioeconomic factors, social factors, and environmental factors were selected as covariates^18,32–34^. For demographic factors, sex assigned at birth (men or women) and age (65–69, 70–74, 75–79, 80–84, or ≥ 85 years) were selected. For health status, body mass index (< 18.5, 18.5–24.9, or ≥ 25 kg/m^2^), current disease (any disease or none), and walking time (< 60 or ≥ 60 min/day) were selected. Regarding current disease status, participants who answered “no” to having any disease under treatment or with lasting aftereffects were classified as having no current disease, whereas all others were classified as having at least one current disease. Subjective cognitive complaints (SCCs) were also included because dementia is associated with depression^5^, and cognitive decline is known to alter sensitivity to abnormal temperatures^35^. SCCs were assessed using three questions from the Kihon Checklist^36^: (1) “Do your family or friends point out your memory loss?” (2) “Are you able to look up phone numbers and make calls yourself?” (3) “Do you find yourself not knowing today’s date?” At least one response of “yes” to the first or third question or “no” to the second question was considered indicative of SCC^37^. For socioeconomic factors, educational attainment (≤ 9, 10–12, or ≥ 13 years), equivalent income (< 2.00, 2.00–3.99, or ≥ 4.00 million JPY)**,** and wealth (total assets of the household including savings, estate and stocks; < 5.00, 5.00–9.99, 10.00–49.99, or ≥ 50.00 million JPY) were selected since these are strongly related to both housing environment and depressive symptoms. The equivalent income was calculated by dividing the household income by the square root of the number of household members. For social factor, marital status (married, widowed, separated, unmarried, or others) was selected. For environmental factors, duration of residence (≤ 5, 5–9, 10–19, 20–29, 30–39, 40–49, or ≥ 50 years), house type (owned house, public rental house, private rental house, or others), and region were selected. House type was included since it has been reported to be associated with mortality risk^32^.

### Statistical analysis

To evaluate the association between perceived indoor cold or heat and depressive symptoms, we employed Poisson regression with sandwich estimators for standard errors and estimated prevalence ratios (PRs) and 95% confidence intervals (CIs). We built the following three models: the crude model (unadjusted), model 1 (adjusted for sex and age), and model 2 (adjusted for all covariates). For the independent variable and covariates, we checked for collinearity based on variance inflation factor^38^ and confirmed no higher multicollinearity among the variables. Under the assumption that the data were missing at random, we conducted multiple imputation by chained equations (MICE) including all variables used in the analysis to address potential selection bias, generating 20 imputed datasets^39,40^. To diagnose convergence, we checked whether there were large variations between each imputation dataset or bias in the patterns. The estimates of each imputed dataset were combined using Rubin’s rules. For the sensitivity analysis, we conducted a complete case analysis to confirm the consistency of the results of multiple imputation data. Additionally, we conducted stratified analyses by geographical regions (each has a distinct climate: Hokkaido, Tohoku, Kanto, Chubu, Kinki, Chugoku, and Kyushu) for the purpose of exploring regional differences in the association between perceived indoor cold or heat and depressive symptoms. Despite including possible confounders in the analysis, it is impossible to deny the possibility that unmeasured confounding factors may have affected the results. Therefore, we have also calculated the E-value of the PR to identify the strength of the unmeasured covariates that affected the estimates. Statistical analyses were performed using Stata MP version 17 (Stata Corp., College Station, TX, USA).

### Ethical approval

The JAGES protocol and informed consent procedure were approved by the Ethics Committee of Chiba University (M10460) and Tohoku University (37582). Informed consent was obtained from all participants. The study was conducted in accordance with the principles of the Declaration of Helsinki.

## Results

Table 1 shows the characteristics of the participants after multiple imputations. Of a total of 17,491 participants selected (age: 74.5 ± 6.1 years [mean ± standard deviation], 49.4% male), 22.8% exhibited depressive symptoms. 5.1% of the participants were living in houses unable to prevent cold or heat, of which 41.7% had depressive symptoms, whereas 94.9% were living in houses that could prevent cold or heat, of which 21.8% had depressive symptoms. The participants’ characteristics before multiple imputation are presented in Supplementary Table 1. While post-imputation and pre-imputation data had similar distributions, relatively high proportions of missing data were observed in covariates such as equivalent income (10.7%) and wealth (16.0%).

**Table 1:** Characteristics of study participants after multiple imputation (n = 17,491).

	Total		Depressive symptoms			
			No (n = 13,501; 77.2%)		Yes (n = 3,990; 22.8%)	
	n	%	n	%	n	%
Indoor thermal environment						
Not cold or hot	16,607	94.9	12,986	78.2	3,621	21.8
Cold or hot	884	5.1	515	58.3	369	41.7
Sex						
Men	8,643	49.4	6,673	77.2	1,970	22.8
Women	8,848	50.6	6,828	77.2	2,020	22.8
Age (years)						
65–69	4,239	24.2	3,238	76.4	1,001	23.6
70–74	5,374	30.7	4,213	78.4	1,161	21.6
75–79	4,041	23.1	3,161	78.2	880	21.8
80–84	2,620	15.0	1,999	76.3	621	23.7
≥ 85	1,217	7.0	890	73.1	327	26.9
BMI						
< 18.5	1,304	7.5	930	71.4	374	28.6
18.5–24.9	12,195	69.7	9,490	77.8	2,705	22.2
≥ 25	3,992	22.8	3,081	77.2	911	22.8
Educational attainment (years of education)						
≤ 9	3,466	19.8	2,466	71.1	1,000	28.9
10–12	7,658	43.8	5,900	77.1	1,757	22.9
≥ 13	6,207	35.5	5,022	80.9	1,186	19.1
Others	160	0.9	113	70.6	47	29.4
Equivalent income (million JPY)						
< 2.00	8,456	48.3	6,045	71.5	2,411	28.5
2.00–3.99	6,831	39.1	5,544	81.2	1,287	18.8
≥ 4.00	2,204	12.6	1,912	86.8	292	13.2
Wealth (million JPY)						
< 5.00	4,730	27.0	3,226	68.2	1,504	31.8
5.00–9.99	2,823	16.1	2,102	74.4	722	25.6
10.00–49.99	7,309	41.8	5,909	80.9	1,399	19.1
≥ 50.00	2,629	15.0	2,264	86.1	365	13.9
Marital status						
Married	13,008	74.4	10,356	79.6	2,652	20.4
Widowed	2,978	17.0	2,198	73.8	781	26.2
Separated	849	4.9	555	65.4	294	34.6
Unmarried	568	3.2	337	59.3	231	40.7
Others	88	0.5	55	63.0	32	37.0
Subjective cognitive complaints						
No	11,963	68.4	9,840	82.2	2,123	17.8
Yes	5,528	31.6	3,661	66.2	1,867	33.8
Walking time						
< 60 min	10,959	62.7	8,124	74.1	2,835	25.9
≥ 60 min	6,532	37.3	5,377	82.3	1,155	17.7
Current disease						
No	3,366	19.2	2,802	83.2	564	16.8
Yes	14,125	80.8	10,699	75.7	3,426	24.3
House type						
Owned house	15,862	90.7	12,401	78.2	3,461	21.8
Public rental house	674	3.9	450	66.8	224	33.2
Private rental house	594	3.4	401	67.6	192	32.4
Others	361	2.1	249	68.8	113	31.2
Duration of residence (years)						
< 5	515	2.9	363	70.4	153	29.6
5–9	603	3.4	448	74.2	155	25.8
10–19	1,460	8.3	1,091	74.7	369	25.3
20–29	2,076	11.9	1,627	78.4	449	21.6
30–39	2,829	16.2	2,198	77.7	631	22.3
40–49	4,048	23.1	3,155	77.9	893	22.1
≥ 50	5,960	34.1	4,619	77.5	1,340	22.5

JPY, Japanese yen.

Table 2 presents the results of the modified Poisson regression analysis. A significant association between indoor cold or heat and depressive symptoms was observed in the crude model (PR = 1.91, 95% CI = 1.76–2.08). After adjusting for covariates, the association remained significant (models 1 & 2). In model 2, the participants living in houses that could not prevent cold or heat had 1.57 (95% CI = 1.45–1.71) times higher prevalence of depressive symptoms than those living in houses that could prevent cold and heat. E-value for the adjusted PR was 2.52. Supplementary Table 2 presents similar results obtained from the complete case analysis (Model 2: PR = 1.60; 95% CI = 1.45–1.76).

**Table 2:** Association between indoor cold or heat and depressive symptoms (n = 17,491).

Indoor thermal environment	Crude model	Model 1^a^	Model 2^b^	
	PR (95% CI)	Adjusted PR (95% CI)	Adjusted PR (95% CI)	E-value^c^ of the adjusted PR from Model 2
Not cold/hot	1.00 (Ref.)	1.00 (Ref.)	1.00 (Ref.)	
Cold/hot	1.91 (1.76–2.08)	1.92 (1.77–2.09)	1.57 (1.45–1.71)	2.52

^a^Adjusted for age and sex.

^b^Adjusted for sex, age, body mass index, educational attainment, income, wealth, marital status, subjective cognitive complaints, walking time, current disease, house type, and duration of residence.

^c^E-value presents the minimum strength of association on the risk ratio scale that an unmeasured confounder would need to have with both exposure and outcome to fully explain away the observed association conditional on included covariates.

Abbreviation: PR, prevalence ratio; 95% CI, 95% confidence intervals.

In the stratified analysis by geographical regions, a significant association between perceived indoor cold or heat and depressive symptoms was observed in those living in the following regions: Tohoku (PR = 1.58, 95% CI = 1.31–1.90), Kanto (PR = 1.64, 95% CI = 1.34–2.02), Chubu (PR = 1.57, 95% CI = 1.36–1.81), Kinki (PR = 1.43, 95% CI = 1.03–2.00), Chugoku (PR = 1.35, 95% CI = 1.01–1.78), and Kyushu (PR = 1.80, 95% CI = 1.36–2.41), in order from north to south (Supplementary Table 3). No significant association was observed in those living in Hokkaido (PR = 1.37, 95% CI = 0.93–2.03), the northernmost region with the coldest climate.

## Discussion

This cross-sectional study of independent older adults demonstrated an association between perceived indoor cold or heat and depressive symptoms. The prevalence ratio was 1.91 in the crude model and 1.57 in model 2, which adjusted for all covariates. This indicates that 37% of the excess prevalence ratio was explained by the covariates included as potential confounders. In the stratified analysis by geographical region, a significant association was observed in all areas except Hokkaido, the northernmost region, with the strongest association found in Kyushu, the southernmost region. Since depressive symptoms are a well-established predictive factor for depression, our findings imply an association between perceived indoor cold or heat and depression^41^.

Our findings are consistent with previous studies regarding indoor cold, but also suggest a potential association between indoor heat and depression. In terms of indoor cold, previous studies have shown that living in cold housing significantly increases the risk of depression^42^. The inability to adequately warm homes, known as energy poverty, has been reported to increase the likelihood of depression/anxiety by about 50%^43^. Indoor cold is known to be a more serious issue in regions with mild winters than in those with severe winter conditions, due to generally lower levels of insulation. For example, in Europe, winter mortality is greater in countries with milder climates than in those with more severe winter conditions^44^. The lack of significant association observed in Hokkaido is consistent with this previous finding, and could be attributed the highest living room temperatures in Japan due to high insulation levels and the approach of heating the entire building continuously^24,33^. Regarding indoor heat, to the best of our knowledge, there is limited evidence of the effects of perceived indoor heat on depression. The strongest association observed in Kyushu, which has hot and humid summers but mildly cold winters^45^, implies that difficulties with indoor heat in the summer may also contribute to depressive symptoms, although it is impossible to determine if this is solely the effect of indoor heat. We believe this implication is important as it raises concerns that indoor heat caused by global warming may lead to increased depression in the future. Taken together, it could be implied that abnormal indoor thermal environments have effects on depression, especially in mild winter regions and severely hot regions.

Based on previous studies, several mechanisms can be hypothesized to explain the effects of indoor cold or heat on depression. In terms of cold housing, first, high systolic blood pressure^46^ and brain vasoconstriction^47^ caused by a cold environment have been suggested to lead to late-life depression through focal fiber tract disruption, altered functional connectivity, and altered regional brain function^48^. Second, low ambient temperature affects the function of the hippocampus^49^, which is associated with depression^50^. On the other hand, in a hot environment, difficulties with brain cooling, oxygenation and increased blood flow^51^ may contribute to depressive symptoms and mental disorders^52^. Furthermore, hyperthermia increases the permeability of the blood–brain barrier (BBB)^53^, which is known to lead to depression^54^. Both cold and hot indoor environments are known to impair sleep quality^13,16^, which is associated with increased depressive symptoms^55^. It has been reported that bedroom temperatures in Japan are approximately 4 °C lower than living room temperatures^24^, which is possibly leading to sleep disturbances and depressive symptoms.

Our findings suggest that improving thermal environments, even in mild winter regions, is necessary to prevent depression among community-dwelling, functionally independent older adults. Possible approaches include thermal insulation, heat supply, air-conditioning, ventilation^56,57^, and relocation. However, the implementation and running costs of heating, air-conditioning, and ventilation systems can be prohibitively expensive. While this population has the autonomy to consider relocation, this option should be approached cautiously, as it is known to lead to relocation stress syndrome (RSS), characterized by confusion, depression, anxiety, apprehension, and loneliness in older adults^58,59^. Moreover, ageing in place has been reported to improve the QOL in low-income people^60,61^. Thus, insulation, which is associated with health benefits and reductions in healthcare expenditures^42,62^, could be considered a potentially effective and practical approach to improving the indoor thermal environment. Importantly, low-income individuals are known to be at higher risk of heat-related mortality^63^ because they are more likely to live in poorly insulated homes and less likely to afford insulation improvements. As such, public support for housing improvements among functionally independent older adults, especially those with low-income, is critical; otherwise, improvements in insulation could lead to further inequalities^8^.

Several limitations of this study should be acknowledged. First, reverse causation is a potential issue since this is a cross-sectional study. There is a possibility that depression leads to living in excessively cold or hot housing, contrary to our hypothesis. A cross-sectional design was the only option because the question item used to assess the exposure variable (“unable to prevent cold or heat”) was first introduced in the 2022 survey. As longitudinal data will become available from the 2025 survey onward, future cohort studies are expected to confirm the temporal relationship between indoor thermal environments and depressive symptoms. Second, the question option used as the independent variable, “Unable to prevent heat or cold,” cannot distinguish between heat and cold. Thus, it is impossible to determine whether the participants are struggling with indoor cold, indoor heat, or both. As this survey was conducted in the winter months (November–December), participants’ responses are more likely to reflect indoor cold rather than indoor heat. Although we inferred which of them might be the risk of depression by conducting stratified analysis based on geographical regions with distinct climates, it is necessary to measure indoor thermal environment using separate options for clearer insight. Third, this independent variable is binary and subjective; thus, there is no information on the extent to which the participants are affected by the abnormal thermal environment, or the actual indoor temperature. Subjective measures, such as the question used in this study, can capture perceived needs and reflect combined effects of temperature, housing conditions, and preferences^18^. However, responses may vary between occupants of the same house. Objective measures, although more consistent, require significant resources, such as cost and effort^64^, and may not reflect time spent in specific rooms. Given the gap between perceived coldness and measured indoor temperatures^33^, and that older adults may be less sensitive to cold yet more vulnerable to its health impacts^33^, future studies should include both subjective and objective measures to validate and expand upon these findings. Fourth, there is a possibility of measurement bias since both the independent and dependent variables are self-reported. However, the dependent variable, the GDS-15, has been validated in previous studies and is widely used as a screening tool for depressive symptoms in older adults. While the independent variable has not been formally validated, potential misclassification is likely to be non-differential and would thus bias the results toward the null. Fifth, our data does not reflect a nationally representative sample because it only includes community-dwelling, functionally independent older adults. Although the 71 participating municipalities were selected to include all regions of the country, the sample does not reflect the balance between urban and rural areas. Sixth, unmeasured confounding may have affected the results. To assess the potential influence of unmeasured confounders, we calculated E-value of the prevalence ratio of depressive symptoms. The obtained E-value was 2.52, indicating that there is an extremely low possibility of unmeasured confounding factors that are strongly associated with both independent and dependent variables.

## Conclusion

The present study revealed that perceived indoor cold or heat exposure is associated with greater prevalence of depressive symptoms. Further research is expected to investigate the impact of improving the indoor thermal environment, such as by installing insulation, on the prevention of depression.

## Supplementary Information

Supplementary Information.

## Data Availability

The datasets used in this study from JAGES are not publicly available because of ethical and legal restrictions. However, the datasets used in this study are available from the corresponding author upon reasonable request.

## References

[1] Wilkinson, P., Ruane, C. & Tempest, K. Depression in older adults. *BMJ* **363**, k4922. 10.1136/bmj.k4922 (2018). 30487197 10.1136/bmj.k4922

[2] GBD 2021 Diseases and Injuries Collaborators. Global incidence, prevalence, years lived with disability (YLDs), disability-adjusted life-years (DALYs), and healthy life expectancy (HALE) for 371 diseases and injuries in 204 countries and territories and 811 subnational locations, 1990–2021: a systematic analysis for the Global Burden of Disease Study 2021. *Lancet*. 2024;403(10440):2133–2161. 10.1016/S0140-6736(24)00757-8 10.1016/S0140-6736(24)00757-8 PMC11122111 38642570

[3] Rodda, J., Walker, Z. & Carter, J. Depression in older adults. *BMJ* **343**(sep28 1), d5219. 10.1136/bmj.d5219 (2011). 21957206 10.1136/bmj.d5219

[4] Soysal, P. et al. Relationship between depression and frailty in older adults: A systematic review and meta-analysis. *Ageing Res. Rev.* **36**, 78–87. 10.1016/j.arr.2017.03.005 (2017). 28366616 10.1016/j.arr.2017.03.005

[5] Diniz, B. S., Butters, M. A., Albert, S. M., Dew, M. A. & Reynolds, C. F. 3rd. Late-life depression and risk of vascular dementia and Alzheimer’s disease: Systematic review and meta-analysis of community-based cohort studies. *Br. J. Psychiatry.* **202**(5), 329–335. 10.1192/bjp.bp.112.118307 (2013). 23637108 10.1192/bjp.bp.112.118307 PMC3640214

[6] Tham, S., Thompson, R., Landeg, O., Murray, K. A. & Waite, T. Indoor temperature and health: A global systematic review. *Public Health* **179**, 9–17. 10.1016/j.puhe.2019.09.005 (2020). 31707154 10.1016/j.puhe.2019.09.005

[7] Janssen, H. et al. Cold indoor temperatures and their association with health and well-being: A systematic literature review. *Public Health* **224**, 185–194. 10.1016/j.puhe.2023.09.006 (2023). 37820536 10.1016/j.puhe.2023.09.006

[8] World Health Organization. WHO Housing and Health Guidelines. Accessed November 5, 2024. https://iris.who.int/bitstream/handle/10665/276001/9789241550376-eng.pdf

[9] Shiue, I. Cold homes are associated with poor biomarkers and less blood pressure check-up: English Longitudinal Study of Ageing, 2012–2013. *Environ. Sci. Pollut. Res. Int.* **23**(7), 7055–7059. 10.1007/s11356-016-6235-y (2016). 26873825 10.1007/s11356-016-6235-y PMC4820485

[10] Umishio, W. et al. Electrocardiogram abnormalities in residents in cold homes: A cross-sectional analysis of the nationwide Smart Wellness Housing survey in Japan. *Environ. Health Prev. Med.* **26**(1), 104. 10.1186/s12199-021-01024-1 (2021). 34641787 10.1186/s12199-021-01024-1 PMC8513347

[11] Saeki, K., Obayashi, K. & Kurumatani, N. Platelet count and indoor cold exposure among elderly people: A cross-sectional analysis of the HEIJO-KYO study. *J. Epidemiol.* **27**(12), 562–567. 10.1016/j.je.2016.12.018 (2017). 28645521 10.1016/j.je.2016.12.018 PMC5623037

[12] Zhao, H., Jivraj, S. & Moody, A. “My blood pressure is low today, do you have the heating on?” The association between indoor temperature and blood pressure. *J. Hypertens.* **37**(3), 504–512. 10.1097/HJH.0000000000001924 (2019). 30134311 10.1097/HJH.0000000000001924

[13] Saeki, K., Obayashi, K., Tone, N. & Kurumatani, N. A warmer indoor environment in the evening and shorter sleep onset latency in winter: The HEIJO-KYO study. *Physiol. Behav.* **149**, 29–34. 10.1016/j.physbeh.2015.05.022 (2015). 26004170 10.1016/j.physbeh.2015.05.022

[14] Lindemann, U. et al. Effect of cold indoor environment on physical performance of older women living in the community. *Age Ageing* **43**(4), 571–575. 10.1093/ageing/afu057 (2014). 24855113 10.1093/ageing/afu057

[15] Sutton-Klein, J., Moody, A., Hamilton, I. & Mindell, J. S. Associations between indoor temperature, self-rated health and socioeconomic position in a cross-sectional study of adults in England. *BMJ Open* **11**(2), e038500. 10.1136/bmjopen-2020-038500 (2021). 33622938 10.1136/bmjopen-2020-038500 PMC7907859

[16] van Loenhout, J. A. F. et al. The effect of high indoor temperatures on self-perceived health of elderly persons. *Environ. Res.* **146**, 27–34. 10.1016/j.envres.2015.12.012 (2016). 26710340 10.1016/j.envres.2015.12.012

[17] O’Lenick, C. R. et al. A case-crossover analysis of indoor heat exposure on mortality and hospitalizations among the elderly in Houston, Texas. *Environ. Health Perspect.* **128**(12), 127007. 10.1289/EHP6340 (2020). 33300819 10.1289/EHP6340 PMC7727721

[18] Clair, A. & Baker, E. Cold homes and mental health harm: Evidence from the UK Household Longitudinal Study. *Soc. Sci.Med.* **314**(115461), 115461. 10.1016/j.socscimed.2022.115461 (2022). 36327633 10.1016/j.socscimed.2022.115461

[19] Chimed-Ochir, O. et al. Effect of housing condition on quality of life. *Indoor Air* **31**(4), 1029–1037. 10.1111/ina.12819 (2021). 33739475 10.1111/ina.12819

[20] Ahrentzen, S., Erickson, J. & Fonseca, E. Thermal and health outcomes of energy efficiency retrofits of homes of older adults. *Indoor Air* **26**(4), 582–593. 10.1111/ina.12239 (2016). 26249033 10.1111/ina.12239

[21] Padhy, S. K., Sarkar, S., Panigrahi, M. & Paul, S. Mental health effects of climate change. *Indian J. Occup. Environ. Med.* **19**(1), 3–7. 10.4103/0019-5278.156997 (2015). 26023264 10.4103/0019-5278.156997 PMC4446935

[22] The Present Condition of Existing Housing Stock. Ministry of Land, Infrastructure, Transport and Tourism (in Japanese). Accessed 5 Nov 2024. https://www.mlit.go.jp/common/001105108.pdf

[23] Murakami, S. et al. Overview of energy consumption and GHG mitigation technologies in the building sector of Japan. *Energy Effic.* **2**(2), 179–194. 10.1007/s12053-008-9040-8 (2009).

[24] Umishio, W. et al. Disparities of indoor temperature in winter: A cross-sectional analysis of the Nationwide Smart Wellness Housing Survey in Japan. *Indoor Air* **30**(6), 1317–1328. 10.1111/ina.12708 (2020). 32573794 10.1111/ina.12708 PMC7689703

[25] Kim, Y. et al. Enhancing health resilience in Japan in a changing climate. *Lancet Reg. Health West Pac.* **40**(100970), 100970. 10.1016/j.lanwpc.2023.100970 (2023). 38116496 10.1016/j.lanwpc.2023.100970 PMC10730320

[26] Emergency medical evacuations due to heat stroke in 2024 (May-September) (in Japanese). Ministry of Internal Affairs and Communications. October 29, 2024. Accessed 30 April 2025. https://www.fdma.go.jp/disaster/heatstroke/items/r6/heatstroke_nenpou_r6.pdf

[27] Matz, C. J. et al. Effects of age, season, gender and urban-rural status on time-activity: Canadian Human Activity Pattern Survey 2 (CHAPS 2). *Int. J. Environ. Res. Public Health.* **11**(2), 2108–2124. 10.3390/ijerph110202108 (2014). 24557523 10.3390/ijerph110202108 PMC3945588

[28] Kondo, K. Progress in aging epidemiology in Japan: The JAGES project. *J. Epidemiol.* **26**(7), 331–336. 10.2188/jea.JE20160093 (2016). 27349200 10.2188/jea.JE20160093 PMC4919477

[29] Malakouti, S. K., Fatollahi, P., Mirabzadeh, A., Salavati, M. & Zandi, T. Reliability, validity and factor structure of the GDS-15 in Iranian elderly. *Int. J. Geriatr. Psychiatry.* **21**(6), 588–593. 10.1002/gps.1533 (2006). 16783767 10.1002/gps.1533

[30] Herrmann, N. et al. A validation study of the Geriatric Depression Scale short form. *Int. J. Geriatr. Psychiatry* **11**(5), 457–460. 10.1002/(SICI)1099-1166(199605)11:5%3c457::AID-GPS325%3e3.0.CO;2-2 (1996).

[31] Pocklington, C., Gilbody, S., Manea, L. & McMillan, D. The diagnostic accuracy of brief versions of the Geriatric Depression Scale: A systematic review and meta-analysis: Geriatric Depression Scale diagnostic accuracy. *Int. J. Geriatr. Psychiatry.* **31**(8), 837–857. 10.1002/gps.4407 (2016). 26890937 10.1002/gps.4407

[32] Koga, C. et al. Living in public rental housing is healthier than private rental housing a 9-year cohort study from Japan Gerontological Evaluation Study. *Sci. Rep.* **14**(1), 7547. 10.1038/s41598-024-58244-y (2024). 38555321 10.1038/s41598-024-58244-y PMC10981673

[33] Umishio, W. et al. Spatial and temporal indoor temperature differences at home and perceived coldness in winter: A cross-sectional analysis of the nationwide Smart Wellness Housing survey in Japan. *Environ. Int.* **186**, 108630. 10.1016/j.envint.2024.108630 (2024). 38593691 10.1016/j.envint.2024.108630

[34] Nishida, M., Hanazato, M., Koga, C. & Kondo, K. Association between Proximity of the Elementary School and Depression in Japanese Older Adults: A Cross-Sectional Study from the JAGES 2016 Survey. *Int. J. Environ. Res. Public Health.* **18**(2), 1. 10.3390/ijerph18020500 (2021). 10.3390/ijerph18020500 PMC7826926 33435418

[35] Fletcher, P. D. et al. Pain and temperature processing in dementia: A clinical and neuroanatomical analysis. *Brain* **138**(Pt 11), 3360–3372. 10.1093/brain/awv276 (2015). 26463677 10.1093/brain/awv276 PMC4620514

[36] Satake, S. et al. Validity of the Kihon Checklist for assessing frailty status: Kihon Checklist as frailty scale. *Geriatr. Gerontol. Int.* **16**(6), 709–715. 10.1111/ggi.12543 (2016). 26171645 10.1111/ggi.12543

[37] Kiuchi, S. et al. Longitudinal association between oral status and cognitive decline using fixed-effects analysis. *J. Epidemiol.* **32**(7), 330–336. 10.2188/jea.JE20200476 (2022). 33518591 10.2188/jea.JE20200476 PMC9189315

[38] Sofer, T. et al. Variant-specific inflation factors for assessing population stratification at the phenotypic variance level. *Nat. Commun.* **12**(1), 3506. 10.1038/s41467-021-23655-2 (2021). 34108454 10.1038/s41467-021-23655-2 PMC8190158

[39] Li, P., Stuart, E. A. & Allison, D. B. Multiple imputation: A flexible tool for handling missing data. *JAMA* **314**(18), 1966. 10.1001/jama.2015.15281 (2015). 26547468 10.1001/jama.2015.15281 PMC4638176

[40] Lee, K. J. et al. Framework for the treatment and reporting of missing data in observational studies: The Treatment And Reporting of Missing data in Observational Studies framework. *J. Clin. Epidemiol.* **134**, 79–88. 10.1016/j.jclinepi.2021.01.008 (2021). 33539930 10.1016/j.jclinepi.2021.01.008 PMC8168830

[41] Shin, C. et al. Usefulness of the 15-item geriatric depression scale (GDS-15) for classifying minor and major depressive disorders among community-dwelling elders. *J. Affect Disord.* **259**, 370–375. 10.1016/j.jad.2019.08.053 (2019). 31470180 10.1016/j.jad.2019.08.053

[42] Mishra, S. R. et al. The total health gains and cost savings of eradicating cold housing in Australia. *Soc. Sci. Med.* **334**, 115954. 10.1016/j.socscimed.2023.115954 (2023). 37672848 10.1016/j.socscimed.2023.115954

[43] Bentley, R., Daniel, L., Li, Y., Baker, E. & Li, A. The effect of energy poverty on mental health, cardiovascular disease and respiratory health: A longitudinal analysis. *Lancet Reg. Health West Pac.* **35**(100734), 100734. 10.1016/j.lanwpc.2023.100734 (2023). 37424688 10.1016/j.lanwpc.2023.100734 PMC10326697

[44] Group TE. Cold exposure and winter mortality from Ischaemic heart disease, cerebrovascular disease, respiratory disease, and all causes in warm and cold regions of Europe. *The Lancet* **349**(9062), 1341–1346. 10.1016/s0140-6736(96)12338-2 (1997). 9149695

[45] Ishimaru, M. et al. Effects of different winter climates in Japan on body composition of young Thoroughbreds in training. *J. Vet. Med. Sci.* **84**(12), 1585–1594. 10.1292/jvms.22-0378 (2022). 36244743 10.1292/jvms.22-0378 PMC9791233

[46] Umishio, W. et al. Role of housing in blood pressure control: a review of evidence from the Smart Wellness Housing survey in Japan. *Hypertens Res.* **46**(1), 9–18. 10.1038/s41440-022-01060-6 (2023). 36224288 10.1038/s41440-022-01060-6 PMC9747607

[47] Muller, M. D. et al. Acute cold exposure and cognitive function: Evidence for sustained impairment. *Ergonomics* **55**(7), 792–798. 10.1080/00140139.2012.665497 (2012). 22506538 10.1080/00140139.2012.665497 PMC3375336

[48] Taylor, W. D., Aizenstein, H. J. & Alexopoulos, G. S. The vascular depression hypothesis: Mechanisms linking vascular disease with depression. *Mol. Psychiatry.* **18**(9), 963–974. 10.1038/mp.2013.20 (2013). 23439482 10.1038/mp.2013.20 PMC3674224

[49] Carrettiero, D. C., Santiago, F. E., Motzko-Soares, A. C. P. & Almeida, M. C. Temperature and toxic Tau in Alzheimer’s disease: New insights. *Temperature (Austin).* **2**(4), 491–498. 10.1080/23328940.2015.1096438 (2015). 27227069 10.1080/23328940.2015.1096438 PMC4843920

[50] Sheline, Y. I. Depression and the hippocampus: Cause or effect?. *Biol. Psychiatry.* **70**(4), 308–309. 10.1016/j.biopsych.2011.06.006 (2011). 21791257 10.1016/j.biopsych.2011.06.006 PMC3733566

[51] Lõhmus, M. Possible biological mechanisms linking mental health and heat-A contemplative review. *Int. J. Environ. Res. Public Health.* **15**(7), 1515. 10.3390/ijerph15071515 (2018). 30021956 10.3390/ijerph15071515 PMC6068666

[52] Fischer, S. et al. Emerging effects of temperature on human cognition, affect, and behaviour. *Biol. Psychol.* **189**(108791), 108791. 10.1016/j.biopsycho.2024.108791 (2024). 38599369 10.1016/j.biopsycho.2024.108791

[53] Sharma, H. S. & Hoopes, P. J. Hyperthermia induced pathophysiology of the central nervous system. *Int. J. Hyperthermia.* **19**(3), 325–354. 10.1080/0265673021000054621 (2003). 12745974 10.1080/0265673021000054621

[54] Medina-Rodriguez, E. M. & Beurel, E. Blood brain barrier and inflammation in depression. *Neurobiol. Dis.* **175**(105926), 105926. 10.1016/j.nbd.2022.105926 (2022). 36375722 10.1016/j.nbd.2022.105926 PMC10035601

[55] Hu, Z. et al. Association between poor sleep quality and depression symptoms among the elderly in nursing homes in Hunan province, China: A cross-sectional study. *BMJ Open* **10**(7), e036401. 10.1136/bmjopen-2019-036401 (2020). 32665347 10.1136/bmjopen-2019-036401 PMC7359068

[56] Breysse, J., Dixon, S. L., Jacobs, D. E., Lopez, J. & Weber, W. Self-reported health outcomes associated with green-renovated public housing among primarily elderly residents. *J. Public Health Manag. Pract.* **21**(4), 355–367. 10.1097/PHH.0000000000000199 (2015). 25679773 10.1097/PHH.0000000000000199

[57] Gasparrini, A. et al. Mortality risk attributable to high and low ambient temperature: A multicountry observational study. *Lancet* **386**(9991), 369–375. 10.1016/S0140-6736(14)62114-0 (2015). 26003380 10.1016/S0140-6736(14)62114-0 PMC4521077

[58] Mallick, M. & Whipple, T. Validity of the nursing diagnosis of relocation stress syndrome. *Nurs. Res.* **49**(2), 97–100. 10.1097/00006199-200003000-00006 (2000). 10768586 10.1097/00006199-200003000-00006

[59] Zeng, W., Wu, Z., Schimmele, C. M. & Li, S. Mass relocation and depression among seniors in China. *Res. Aging.* **37**(7), 695–718. 10.1177/0164027514551178 (2015). 25651588 10.1177/0164027514551178

[60] Rahmati, M. et al. The effectiveness of community ageing in place, advancing better living for elders as a biobehavioural environmental approach for disability among low-income older adults: A systematic review and meta-analysis. *Age Ageing.* **52**(4), 1. 10.1093/ageing/afad053 (2023). 10.1093/ageing/afad053 37078754

[61] Morley, J. E. Aging in place. *J. Am. Med. Dir Assoc.* **13**(6), 489–492. 10.1016/j.jamda.2012.04.011 (2012). 22682696 10.1016/j.jamda.2012.04.011

[62] Preval, N., Keall, M., Telfar-Barnard, L., Grimes, A. & Howden-Chapman, P. Impact of improved insulation and heating on mortality risk of older cohort members with prior cardiovascular or respiratory hospitalisations. *BMJ Open* **7**(11), e018079. 10.1136/bmjopen-2017-018079 (2017). 29138207 10.1136/bmjopen-2017-018079 PMC5695334

[63] Basu, R. High ambient temperature and mortality: A review of epidemiologic studies from 2001 to 2008. *Environ. Health.* **8**(1), 1–13. 10.1186/1476-069X-8-40 (2009). 19758453 10.1186/1476-069X-8-40 PMC2759912

[64] Kanno, I. et al. Relationship between the housing coldness/warmth evaluation by CASBEE Housing Health Checklist and psychological distress based on TMM Community-based cohort study: A cross-sectional analysis. *Public Health* **208**, 98–104. 10.1016/j.puhe.2022.05.003 (2022). 35738131 10.1016/j.puhe.2022.05.003

