# Supplementary material for: Perceived indoor thermal environment and depressive symptoms among older adults in the Japan Gerontological Evaluation Study
Source: Sci Rep. 2025 Aug 22;15:30871. doi: 10.1038/s41598-025-15922-9 (PMC12373872; doi:10.1038/s41598-025-15922-9)
Supplement: Supplementary file 1 — Supplementary Information. [file 41598_2025_15922_MOESM1_ESM.docx]

*Supplementary material*

**Title: Perceived indoor thermal environment and depressive symptoms among older adults in the Japan Gerontological Evaluation Study**

Table of Contents

[Supplementary Table 1. Characteristics of study participants before multiple imputation (n = 17,491) 2](#_Toc157174232)

[Supplementary Table 2. Association between indoor cold or heat and depressive symptoms: Comparison of complete case (n = 13,226) and multiple imputation analyses (n=17,491) 4](#_Toc157174233)

[Supplementary Table 3. Association between indoor cold or heat and depressive symptoms stratified by region (n = 17,491) 5](#_Toc157174234)

# **Supplementary Table 1. Characteristics of study participants before multiple imputation (n = 17,491)**

|  | Total | | Depressive symptoms | | | |
| --- | --- | --- | --- | --- | --- | --- |
|  |  | | Yes | | No | |
|  | n | % | n | % | n | % |
| **Indoor thermal environment** |  |  |  |  |  |  |
| Not cold or hot | 16,607 | 94.9 | 12,986 | 78.2 | 3,621 | 21.8 |
| Cold or hot | 884 | 5.1 | 515 | 58.3 | 369 | 41.7 |
| **Sex** |  |  |  |  |  |  |
| Men | 8,643 | 49.4 | 6,673 | 77.2 | 1,970 | 22.8 |
| Women | 8,848 | 50.6 | 6,828 | 77.2 | 2,020 | 22.8 |
| **Age (years)** |  |  |  |  |  |  |
| 65–69 | 4,239 | 24.2 | 3,238 | 76.4 | 1,001 | 23.6 |
| 70–74 | 5,374 | 30.7 | 4,213 | 78.4 | 1,161 | 21.6 |
| 75–79 | 4,041 | 23.1 | 3,161 | 78.2 | 880 | 21.8 |
| 80–84 | 2,620 | 15.0 | 1,999 | 76.3 | 621 | 23.7 |
| ≥85 | 1,217 | 7.0 | 890 | 73.1 | 327 | 26.9 |
| **BMI** |  |  |  |  |  |  |
| <18.5 | 1,277 | 7.3 | 912 | 71.4 | 365 | 28.6 |
| 18.5–24.9 | 11,964 | 68.4 | 9,328 | 78.0 | 2,636 | 22.0 |
| ≥25 | 3,926 | 22.4 | 3,035 | 77.3 | 891 | 22.7 |
| Missing | 324 | 1.9 | 226 | 69.8 | 98 | 30.2 |
| **Educational attainment (years of education)** | | |  |  |  |  |
| ≤9 | 3,435 | 19.6 | 2,445 | 71.2 | 990 | 28.8 |
| 10–12 | 7,605 | 43.5 | 5,862 | 77.1 | 1,743 | 22.9 |
| ≥13 | 6,170 | 35.3 | 4,993 | 80.9 | 1,177 | 19.1 |
| Others | 159 | 0.9 | 112 | 70.4 | 47 | 29.6 |
| Missing | 122 | 0.7 | 89 | 73.0 | 33 | 27.0 |
| **Equivalent income (million JPY)** | |  |  |  |  |  |
| <2.00 | 7,460 | 42.7 | 5,342 | 71.6 | 2,118 | 28.4 |
| 2.00–3.99 | 6,157 | 35.2 | 4,999 | 81.2 | 1,158 | 18.8 |
| ≥4.00 | 2,003 | 11.5 | 1,742 | 87.0 | 261 | 13.0 |
| Missing | 1,871 | 10.7 | 1,418 | 75.8 | 453 | 24.2 |
| **Wealth (million JPY)** |  |  |  |  |  |  |
| <5.00 | 3,801 | 21.7 | 2,612 | 68.7 | 1,189 | 31.3 |
| 5.00–9.99 | 2,338 | 13.4 | 1,756 | 75.1 | 582 | 24.9 |
| 10.00–49.99 | 6,248 | 35.7 | 5,076 | 81.2 | 1,172 | 18.8 |
| ≥50.00 | 2,300 | 13.1 | 1,990 | 86.5 | 310 | 13.5 |
| Missing | 2,804 | 16.0 | 2,067 | 73.7 | 737 | 26.3 |
| **Marital status** |  |  |  |  |  |  |
| Married | 12,976 | 74.2 | 10,337 | 79.7 | 2,639 | 20.3 |
| Widowed | 2,965 | 17.0 | 2,191 | 73.9 | 774 | 26.1 |
| Separated | 845 | 4.8 | 553 | 65.4 | 292 | 34.6 |
| Unmarried | 565 | 3.2 | 336 | 59.5 | 229 | 40.5 |
| Others | 87 | 0.5 | 55 | 63.2 | 32 | 36.8 |
| Missing | 53 | 0.3 | 29 | 54.7 | 24 | 45.3 |
| **Subjective cognitive complaint** | |  |  |  |  |  |
| No | 11,860 | 67.8 | 9,756 | 82.3 | 2,104 | 17.7 |
| Yes | 5,478 | 31.3 | 3,630 | 66.3 | 1,848 | 33.7 |
| Missing | 153 | 0.9 | 115 | 75.2 | 38 | 24.8 |
| **Walking time** |  |  |  |  |  |  |
| <60 minutes | 10,761 | 61.5 | 7,984 | 74.2 | 2,777 | 25.8 |
| ≥60 minutes | 6,419 | 36.7 | 5,288 | 82.4 | 1,131 | 17.6 |
| Missing | 311 | 1.8 | 229 | 73.6 | 82 | 26.4 |
| **Current disease** |  |  |  |  |  |  |
| No | 3,264 | 18.7 | 2,717 | 83.2 | 547 | 16.8 |
| Yes | 13,736 | 78.5 | 10,406 | 75.8 | 3,330 | 24.2 |
| Missing | 491 | 2.8 | 378 | 77.0 | 113 | 23.0 |
| **House type** |  |  |  |  |  |  |
| Owned house | 15,540 | 88.8 | 12,158 | 78.2 | 3,382 | 21.8 |
| Public rental house | 658 | 3.8 | 440 | 66.9 | 218 | 33.1 |
| Private rental house | 578 | 3.3 | 391 | 67.6 | 187 | 32.4 |
| Others | 351 | 2.0 | 242 | 68.9 | 109 | 31.1 |
| Missing | 364 | 2.1 | 270 | 74.2 | 94 | 25.8 |
| **Duration of residence (years)** | |  |  |  |  |  |
| <5 | 500 | 2.9 | 353 | 70.6 | 147 | 29.4 |
| 5-9 | 584 | 3.3 | 436 | 74.7 | 148 | 25.3 |
| 10–19 | 1,415 | 8.1 | 1,062 | 75.1 | 353 | 24.9 |
| 20–29 | 2,022 | 11.6 | 1,591 | 78.7 | 431 | 21.3 |
| 30–39 | 2,754 | 15.7 | 2,147 | 78.0 | 607 | 22.0 |
| 40–49 | 3,945 | 22.6 | 3,083 | 78.1 | 862 | 21.9 |
| ≥50 | 5,799 | 33.2 | 4,509 | 77.8 | 1,290 | 22.2 |
| Missing | 472 | 2.7 | 320 | 67.8 | 152 | 32.2 |

JPY, Japanese yen

# **Supplementary Table 2. Association between indoor cold or heat and depressive symptoms: Comparison of complete case (n = 13,226) and multiple imputation analyses (n=17,491)**

| Indoor thermal environment | Crude model |  | Model 1^a^ |  | Model 2^b^ |  |
| --- | --- | --- | --- | --- | --- | --- |
|  | PR (95% CI) | | Adjusted PR (95% CI) | | Adjusted PR (95% CI) | |
|  | Complete case | Multiple imputation | Complete case | Multiple imputation | Complete case | Multiple imputation |
| Not cold/hot | 1.00 (Ref.) | 1.00 (Ref.) | 1.00 (Ref.) | 1.00 (Ref.) | 1.00 (Ref.) | 1.00 (Ref.) |
| Cold/hot | 1.98  (1.80–2.19) | 1.91  (1.76–2.08) | 1.98  (1.79–2.18) | 1.92  (1.77–2.09) | 1.60  (1.45–1.76) | 1.57  (1.45–1.71) |

^a^ Adjusted for age and sex

^b^ Adjusted for sex, age, body mass index, educational attainment, income, wealth, marital status, subjective cognitive complaint, walking time, current disease, house type, and duration of residence.

Abbreviation: PR, prevalence ratio; 95%CI, 95% confidence intervals

**Supplementary Table 3. Association between indoor cold or heat and depressive symptoms stratified by region (n=17,491)**

|  |  | PR (95% CI) |  |  |
| --- | --- | --- | --- | --- |
| Region | Indoor thermal environment | Crude model | Model 1 | Model 2 |
| Hokkaido (n=815) | Not cold/hot | 1.00 (Ref.) | 1.00 (Ref.) | 1.00 (Ref.) |
|  | Cold/hot | 1.54 (1.05–2.24) | 1.55 (1.06–2.28) | 1.37 (0.93–2.03) |
| Tohoku (n=1,820) | Not cold/hot | 1.00 (Ref.) | 1.00 (Ref.) | 1.00 (Ref.) |
|  | Cold/hot | 1.82 (1.51–2.20) | 1.84 (1.53–2.22) | 1.58 (1.31–1.90) |
| Kanto (n=4,139) | Not cold/hot | 1.00 (Ref.) | 1.00 (Ref.) | 1.00 (Ref.) |
|  | Cold/hot | 1.93 (1.57–2.37) | 1.92 (1.56–2.37) | 1.64 (1.34–2.02) |
| Chubu (n=7,163) | Not cold/hot | 1.00 (Ref.) | 1.00 (Ref.) | 1.00 (Ref.) |
|  | Cold/hot | 1.91 (1.66–2.19) | 1.91 (1.66–2.19) | 1.57 (1.36–1.81) |
| Kinki (n=1,557) | Not cold/hot | 1.00 (Ref.) | 1.00 (Ref.) | 1.00 (Ref.) |
|  | Cold/hot | 1.72 (1.24–2.38) | 1.71 (1.23–2.38) | 1.43 (1.03–2.00) |
| Chugoku (n=832) | Not cold/hot | 1.00 (Ref.) | 1.00 (Ref.) | 1.00 (Ref.) |
|  | Cold/hot | 1.74 (1.32–2.30) | 1.74 (1.32–2.29) | 1.35 (1.01–1.78) |
| Kyushu (n=1,165) | Not cold/hot | 1.00 (Ref.) | 1.00 (Ref.) | 1.00 (Ref.) |
|  | Cold/hot | 2.44 (1.80–3.32) | 2.41 (1.78–3.26) | 1.80 (1.36–2.41) |

^a^ Adjusted for age and sex

^b^ Adjusted for sex, age, body mass index, educational attainment, income, wealth, marital status, subjective cognitive complaint, walking time, current disease, house type, and duration of residence.

Abbreviation: PR, prevalence ratio; 95%CI, 95% confidence intervals
